# Improving U.S. Biosafety and Biosecurity: Revisiting Recommendations from the Federal Experts Security Advisory Panel and the Fast Track Action Committee on Select Agent Regulations

**DOI:** 10.1089/apb.2022.0025

**Published:** 2023-03-06

**Authors:** Clint A. Haines, Gigi Kwik Gronvall

**Affiliations:** ^1^ Johns Hopkins Center for Health Security, Baltimore, Maryland, USA.; ^2^ Department of Environmental Health and Engineering, Johns Hopkins Bloomberg School of Public Health, Baltimore, Maryland, USA.

**Keywords:** biosafety, biosecurity, select agents, research governance, policy

## Abstract

**Introduction:**

In response to a series of biosafety incidents in 2014, the White House directed two high-level expert committees to analyze biosafety and biosecurity in U.S. laboratories and make recommendations for work with select agents and toxins. Overall, they recommended 33 actions to address areas related to national biosafety, including promoting a culture of responsibility, oversight, outreach and education, applied biosafety research, incident reporting, material accountability, inspection processes, regulations and guidelines, and determining the necessary number of high-containment laboratories in the United States.

**Methods:**

The recommendations were collected and grouped into categories previously defined by the Federal Experts Security Advisory Panel and the Fast Track Action Committee. Open-source materials were examined to determine what actions had been taken to address the recommendations. The actions taken were compared against the reasoning provided in the committee reports to determine if the concerns were sufficiently addressed.

**Results:**

In this study, we found that 6 recommendations were not addressed and 11 were insufficiently addressed out of 33 total recommended actions.

**Discussion and Conclusion:**

Further work is needed to strengthen biosafety and biosecurity in U.S. laboratories handling regulated pathogens (biological select agents and toxins [BSAT]). These carefully considered recommendations should now be enacted, including determining if there is sufficient high-containment laboratory space for response to a future pandemic, developing a sustained applied biosafety research program to improve our understanding of how high-containment research should be performed, bioethics training to educate the regulated community on the consequences of unsafe practices in BSAT research, and the creation of a no-fault incident reporting system for biological incidents, which may inform and improve biosafety training.

**Significance:**

The work presented in this study is significant because previous incidents that occurred in Federal laboratories highlighted shortcomings in the Federal Select Agent Program and the Select Agent Regulations. Progress was made on implementing recommendations to address the shortcomings, but efforts were lost or forgotten over time. The COVID-19 pandemic has provided a brief window of interest in biosafety and biosecurity, and an opportunity to address these shortcomings to increase readiness for future disease emergencies.

## Introduction

A variety of incidents occurred in 2014 that shined a spotlight on biosafety shortcomings. Within the span of a few months, three separate incidents demonstrated that pathogens were not being handled with appropriate levels of safety and security. The first two incidents involved highly pathogenic influenza virus and anthrax at the Centers for Disease Control and Prevention (CDC).^1,2^ In the third incident, vials containing live smallpox virus (variola major) were found, having been abandoned for decades in a National Institutes of Health (NIH) cold room.^3^ Finding samples of smallpox drew media attention because no laboratories are permitted to have stocks of the virus besides the CDC and Vector Institute in the Russian Federation.

In response to these incidents, the White House initiated immediate “stand downs” for laboratory work across the government to investigate what had occurred.^4^ The White House also called for long-term steps for improving biosafety and biosecurity, establishing a new interagency group to examine the impact of the pathogen regulations on national security, science, and technology.^4^ These initiatives were overseen by two Federal advisory committees, the Federal Experts Security Advisory Panel (FESAP) and Fast Track Action Committee on Select Agent Regulations (FTAC-SAR), which initiated a 3-month and ∼13-month process, respectively, to examine the United States' approach to biosafety and biosecurity and to make specific recommendations for improvement.^4–6^

The FESAP committee and FTAC reviewed biosafety and biosecurity issues regarding biological select agents and toxins (BSAT) specifically, but they are not the final authority on all biosafety and biosecurity issues in the United States. The committees released their recommended actions to address a variety of issues, including promoting a culture of responsibility, oversight, outreach and education, applied biosafety research, incident reporting, material accountability, inspection process, regulations and guidelines, and determining the necessary number of high-containment laboratories in the United States. The recommendations from the FESAP were drafted by a group of Federal experts, and ∼55 subject matter experts participated in a thoughtful and deliberative process, including two public hearings, for the FTAC to provide insight that led to the crafting of their recommendations.^6,7^

In this study, we examined whether the recommended actions were addressed by 2022 and we found that most were not sufficiently addressed. At this time there is a renewed interest in biosafety, due to debates over the origin of SARS-CoV-2.^8^ Although evidence points to a natural and not a laboratory origin, this renewed interest in biosafety is an opportunity to consider and address the thoughtful recommendations made by experts after two stakeholder insight meetings, a Request for Public Comment, and months of deliberation.^5–7,9^

### Biological Select Agents and Toxins

The possession and use of certain biological agents are regulated in the United States. There is a list of regulated pathogens and toxins called “biological select agents and toxins (BSAT),” defined as biological organisms or toxins that can threaten the well-being of animal health or products, plant health or products, or human public health and safety.^10,11^ The possession, transfer, and use of BSAT is regulated by a governmental entity known as the Federal Select Agent Program (FSAP), a collaboration between the CDC's Division of Select Agents and Toxins and the U.S. Department of Agriculture's (USDA) Division of Agricultural Select Agents and Toxins.^11,12^

The FSAP requires a security risk assessment (SRA) for responsible officials, alternate responsible officials, any individual with access to BSAT, and any individual who manages BSAT possession, use, or transfer.^13^ An SRA requires an entity's responsible official to request that an individual be given access to BSAT through a secure information system called eFSAP, the responsible official must generate a new Department of Justice (DOJ) unique identifying number (UIN) for the individual, the individual requiring access must complete form FD-961 and two fingerprint cards, and the responsible official must submit the packet containing the FD-961 form and fingerprint cards to the Federal Bureau of Investigation's Bioterrorism Risk Assessment Group for review.^13^

Other requirements for working with BSAT include undergoing annual laboratory inspections and reporting incidents to the FSAP within a week.^13–16^ Some work is required to be reported immediately and entities are not given 7 days. An APHIS/CDC Form 3: Report of a Release, Loss, or Theft is required whenever a BSAT is spilled or released out of primary containment, when there is a potential exposure, or when there is loss of an agent.^17^ The form still needs to be submitted even if the potential exposure is minimal or if a lost agent is recovered.^17^ These requirements are just a small portion of the actions necessary to obtain and maintain the ability to perform BSAT research.

There are even more requirements for working with Tier 1 BSAT, which are BSAT with the highest risk of deliberate misuse that could lead to mass casualties or severe damage to public safety.^18^ One of these requirements is a suitability assessment designed to ensure the individual requesting access to work with Tier 1 BSAT will not misuse them for malicious purposes.^18^ An entity's suitability program must involve a preaccess screening and ongoing monitoring that includes observation, self-reporting, and peer-reporting.^18^

Despite the existing requirements in place to work with BSAT, three laboratory incidents came to light in the summer of 2014 and led to scrutiny of how select agents and toxins were handled. The first incident occurred at the CDC's Influenza Division in January 2014.^1^ A sample of low pathogenic avian influenza (LPAI) was cross-contaminated with a strain of highly pathogenic avian influenza (HPAI).^1^ The USDA's Southeast Poultry Research Laboratory (SEPRL) requested a sample of the LPAI for research purposes, and the sample was sent in March 2014.^1^ No researchers at SEPRL were exposed to HPAI because all manipulations were performed in Biosafety Level 3 (BSL-3) facilities instead of Biosafety Level 2 (BSL-2) facilities where LPAI are typically handled.^1^

The second incident occurred at the CDC's Roybal Campus in June 2014.^2^ A set of *Bacillus anthracis* samples were inactivated in a BSL-3 facility using a procedure that had not been validated for use with *B. anthracis.*^2^ Improperly inactivated samples were brought into a BSL-2 facility for further experimentation.^19^ As a result, 75 CDC staff members were exposed to anthrax, although thankfully none developed symptomatic disease.^2,19^

The final incident occurred at the NIH's Bethesda campus in July 2014.^3^ A 1954-era cardboard box containing six sealed glass vials of freeze-dried smallpox was found in a cold room that the Food and Drug Administration (FDA) was leasing from the NIH while a laboratory was being shut down to move to a different location.^3^ Further testing, which occurred at a CDC facility in Atlanta, determined two vials still contained viable virus.^3^ No workers or staff members were exposed, but this incident garnered international media attention.^3^

### Renewed Focus on Biosafety and Biosecurity

In response to these biosafety lapses, the Obama Administration issued a memorandum from John Holdren (Assistant to the President for Science and Technology) and Lisa Monaco (Assistant to the President for Homeland Security and Counterterrorism), calling for steps to improve national biosafety and biosecurity.^4^ Short-term steps included a safety stand down for all Federal laboratories and laboratories receiving Federal funding for a review of biosafety and biosecurity best practices, laboratory protocols, and a sweep for improperly stored BSAT.^4^ Long-term steps involved instructing an existing interagency committee to make recommendations on how to improve biosafety and biosecurity and establishing a new interagency group to determine the impact of the select agent regulations (SAR) on national security, science, and technology.^4^

These initiatives were overseen by the FESAP and FTAC, respectively.^4^ Each committee released a series of recommended actions that could improve the nation's biosafety and biosecurity.^20^ The FESAP committee released their recommendations in December 2014, and the FTAC in October 2015.^6,7^ The process for crafting these recommendations involved two meetings where SAR stakeholders were able to provide feedback on the SARs, a Request for Public Comment that was published in the Federal Register, and over a year of careful deliberation around the suggestions.^5–7^ A total of 55 stakeholders attended the 2 feedback meetings and 43 submissions were made to the Request for Public Comment.^6^

This article analyzes the progress in implementing the committees' recommendations through laws, guidance documents, and the development of trainings or programs. In most cases, the recommendations have not been sufficiently addressed. This study uses the analysis of open-source material to determine which recommendations were enacted, determines if the actions taken were sufficient to meet the goals stated in the original plan, and suggests steps that should be taken now to improve biosafety and biosecurity in U.S. laboratories.

## Materials and Methods

The recommendations from the FESAP and FTAC were gathered by reviewing the reports released by each group in response to the memorandum from John Holdren and Lisa Monaco.^6,7^ The recommendations were collected and grouped into nine categories previously defined by the FESAP and FTAC for ease of review and assessment.

Open-source materials were examined to determine what actions, if any, had been taken to address the recommendations made by the FESAP and FTAC. The review process involved searching the current SAR, reviewing previous notices of proposed rulemaking (NPRM) for changes to the SAR, reviewing updates on the websites of the CDC and FSAP, and examining open-source news outlets. Search terms used for this process include 42 CFR 73, BSAT, *Biosafety in Microbiological and Biomedical Laboratories (BMBL)*, BSAT international standards, BSAT safety research standards, FTAC-SAR, FESAP, FESAP recommendations 1.1–2.8, FESAP high-containment laboratory number, FSAP guidance documents, FSAP responsible official, FTAC-SAR recommendations 1–13, Interagency Biorisk Management Group, International Expert Group for Biosafety and Biosecurity Regulation (IEGBBR), and SAR.

The actions taken to address each recommendation were compared against the reasoning provided in the FESAP and FTAC reports to determine if the concerns were sufficiently addressed. An item was determined to be addressed if the recommendation was enacted, insufficiently addressed if the recommendation was partially enacted, and not addressed if the recommendation was not enacted. Further recommendations made in the Results and Discussion section of each category were developed by examining the original intent of each recommendation provided in the FESAP and FTAC reports, reviewing relevant reports and recommendations from additional scholars and organizations in the field, and assessing the former and current BSAT research capacity needs of the United States.

## Results and Discussion

The FESAP and FTAC released a plan to implement their recommendations on how to improve national biosafety and biosecurity in October 2015.^20^ The recommendations and completion status can be viewed in Table 1 and are summarized with next step actions.

**Table 1.** Completion status of recommendations from the Federal Experts Security Advisory Panel and the Fast Track Action Committee on the Select Agent Regulations

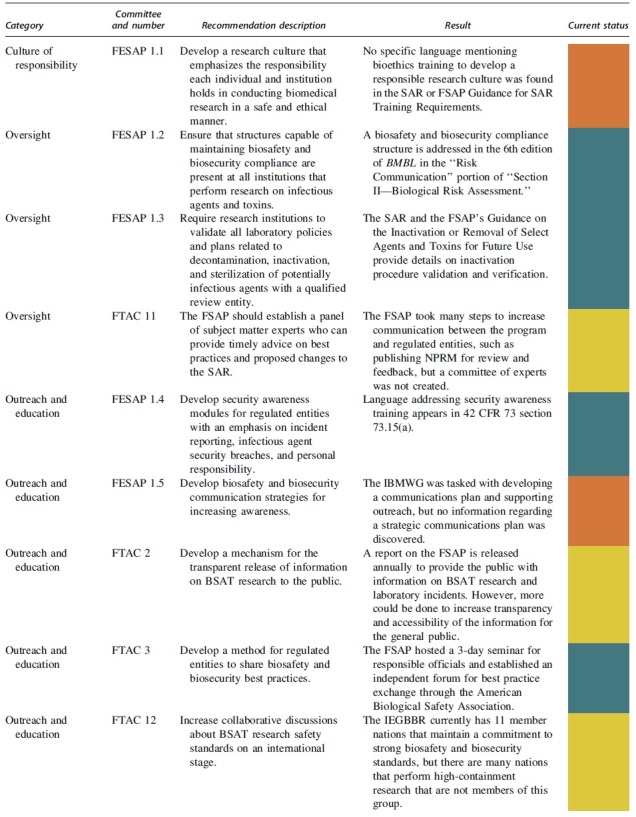 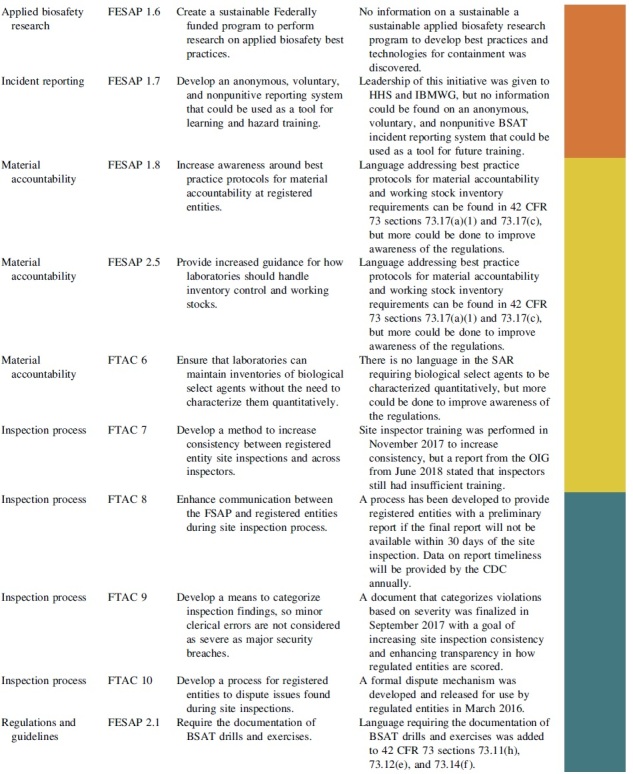 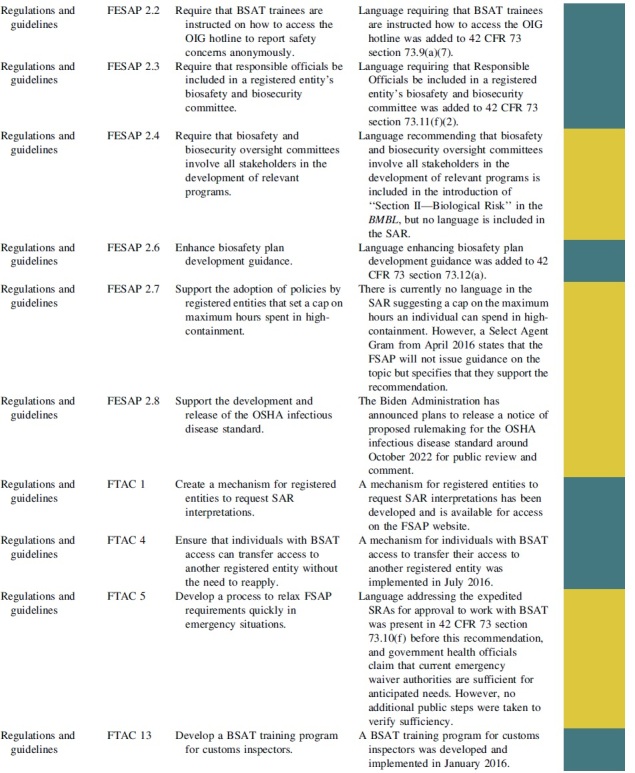 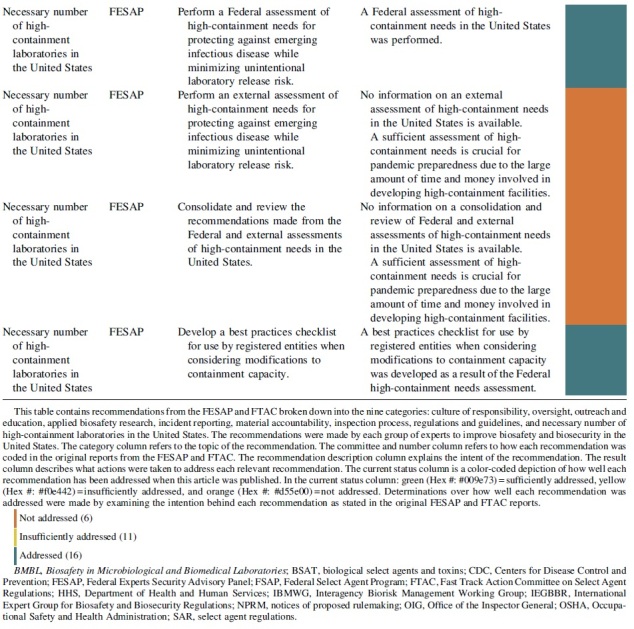

### Culture of Responsibility

Ensuring a “culture of responsibility” is important as the three 2014 laboratory incidents may have been prevented if existing procedures had been followed appropriately.^7^ Plans involved developing training modules to include material on research ethics, legal ramifications of research misconduct, and how research could impact society.^7^ The goal of the plan would be to include the bioethics training modules into existing biosafety and biosecurity training programs to further educate researchers on how misconduct impacts whole communities.^7^

No specific language requiring bioethics training to develop a responsible research culture was found in the SAR or the FSAP's Guidance for SAR Training Requirements document.^21,22^ A culture of responsibility could be further developed by adding language to the SAR, potentially in 42 CFR 73 section 73.15, requiring periodic bioethics training for BSAT researchers. However, efforts should be made to avoid developing a monotonous computer-based training module, and discussion-based training that allows for participation and engagement should be encouraged.^23^

### Oversight

Improving oversight powers for biosafety and biosecurity professionals would help ensure that safety standards and protocols are appropriately designed and that they are being followed by all laboratories. Many research institutions already have biosafety and biosecurity structures, but these recommendations were designed to further support those structures. The plan to implement these recommendations involved ensuring all research institutions had a governance structure to oversee regulatory compliance, developing an entity responsible for validating laboratory protocols related to inactivation of infectious agents, and developing a means for subject matter experts to advise the FSAP.

The CDC/NIH guidelines for biosafety, the 6th edition of *BMBL* in the “Risk Communication” portion of “Section II—Biological Risk Assessment” states that an oversight structure is necessary at institutions that perform work on infectious agents or toxins.^24^ The SAR and FSAP's Guidance on the Inactivation and Removal of Select Agents and Toxins for Future Use provide details on inactivation procedure validation and verification.^21,25^ In addition, NIH guidelines detail inactivation requirements for research involving recombinant or synthetic nucleic acid molecules, and an entity's Institutional Biosafety Committee is charged with reviewing laboratory procedures and practices, including inactivation, with recombinant or synthetic nucleic acid molecules.^26^

The FSAP took steps to increase communication with regulated entities but did not create an expert committee.^27^ The FSAP developed training events for responsible officials, independent forums for regulated entities to share best practices, and published NPRMs to solicit feedback before new regulations are adopted.^27^ According to the original FTAC recommendation, BSAT research oversight could be further improved by re-examining whether the FSAP would benefit from the creation of a committee of external subject matter experts who could advise the program as needed.^20^ A formal peer advisory committee may be able to provide more timely advice in emergency situations.

### Outreach and Education

Increased biosafety and biosecurity awareness within the scientific community would give scientists the tools to prevent mistakes and mitigate potential safety threats.^7^ The plan to improve outreach and education involved the development of security awareness modules for regulated entities, the development of biosafety and biosecurity communication strategies, the transparent release of information on BSAT research to the public, the sharing of best practices between regulated entities, and increasing collaborative discussions on BSAT research safety standards internationally. Implementation of the security awareness modules was addressed in 42 CFR 73 section 73.15(a).^21^

In addition, the FSAP uses an electronic communication system known as Select Agent Grams to disseminate information to the regulated community.^28^ The development of biosafety and biosecurity communication strategies was not completed. The goal of increasing BSAT research transparency with the public was partially completed. As a result of this goal, a report on the FSAP is released annually to provide the public with information on BSAT research and laboratory incidents.^29^ The goal of increasing transparency in BSAT research safety standards among nations was partially completed through an IEGBBR, currently comprising 11 nations that maintain a commitment to strong biosafety and biosecurity standards.^30^

Increasing transparency in BSAT research helps to build trust with the communities that host high-containment laboratories. Improving communication in how incidents are reported would help; 193 reports of select agent release in 2018 may be concerning to individuals without biomedical research experience.^29^ The current infographic that accompanies the FSAP annual report contains information describing how many releases resulted in illness, death, or transmission.^31^ However, providing additional details about types of BSAT release and why they are not always a cause for concern could increase transparency while preventing misinformation.^31,32^

The effort to provide additional details about why certain instances of BSAT release are not always a cause for concern could include information about how accidental needle sticks, or other methods of exposure, can be closely monitored by medical professionals and sometimes treated with postexposure prophylaxis.^33^ The effort could also include information about release events where containment is broken but no one is exposed to the agent, such as when a BSAT containing vial is dropped and broken in a BSL-3 setting where all occupants are wearing appropriate personal protective equipment (PPE).^34^ In this scenario, no workers are exposed to a BSAT due to the appropriate PPE, but an APHIS/CDC Form 3: Report of a Release, Loss, or Theft is still filed.

### Applied Biosafety Research

An applied biosafety research program could investigate new biosafety technologies and laboratory best practices and protocols. An applied biosafety research program may have prevented the *B. anthracis* exposure in 2014 by testing various inactivation protocols for efficacy.^7^ Currently, inactivation protocols are reviewed by an entity's Responsible Official. An applied biosafety research program does not yet exist.

The current lack of funding for applied biosafety research hampers the ability of the scientific community to prevent laboratory accidents and identify cost-effective methods to mitigate risk.^35^ A sustained research program could answer questions about laboratory best practices and potentially improve on current biosafety technologies. A 2019 brief from the Center for Strategic and International Studies estimates that an applied biosafety research program could be started with $10 million.^35^

### Incident Reporting

A nonpunitive BSAT incident reporting system modeled after the Aviation Safety Reporting System (ASRS) could allow lessons to be learned from incidents and near-misses.^36^ No system for an anonymous voluntary nonpunitive BSAT incident reporting system that could allow the regulated community to learn from past mistakes has been created.

### Material Accountability

The plan to improve material accountability awareness and understanding involved increasing awareness around best practice protocols for material accountability, providing increased guidance for how laboratories should handle working stocks, and ensuring that laboratories can maintain biological select agent stocks without quantitative characterization. Best practice protocols for material accountability and working stock inventory requirements can be found in 42 CFR 73 sections 73.17(a)(1) and 73.17(c).^21,28,37^ Material accountability could be further improved by including working stock inventory best practices in the ongoing Responsible Official workshops, in mandatory training for BSAT researchers, and by adding wording to the FSAP Regulatory Interpretations website specifying that nonquantitative record keeping should be encouraged for BSATs.^27,38^

### Inspection Process

Inconsistent site inspections were a problem for regulated entities, as well as a mechanism to dispute inspection findings.^6^ The plan to improve the inspection process involved improving consistency between site inspections, enhancing communication between the FSAP and registered entities, categorizing inspection findings by severity, and developing a site inspection finding dispute mechanism. The recommendation to improve consistency was partially addressed by a training for site inspectors in November 2017, but a report from the Office of the Inspector General (OIG) in June 2018 stated that inspectors still had insufficient training to properly oversee site inspections.^39,40^

The recommendation to enhance communication between the FSAP and registered entities was addressed, and a process has been developed to provide more timely reports.^40^ Categorizing inspection findings by severity was addressed in September 2017.^40^ A formal dispute mechanism was developed and released for use by regulated entities in March 2016.^40^ Building on the recommendation from the FTAC, the consistency of site inspections may benefit from a sustained semiannual training program that collects and discusses inconsistency reports. The program could allow the site inspectors to “war game” previous inspections and develop methods to improve consistency in an interactive environment.

### Regulations and Guidelines

The FESAP committee and FTAC agreed that BSAT research handling was well regulated, but changes could make the FSAP more effective. The plan to improve the FSAP regulations and guidelines involved requiring the documentation of BSAT drills and exercises, training on how to access the OIG anonymous hotline to report safety concerns, requiring that responsible officials be included in biosafety and biosecurity committees, suggesting the adoption of policies that set a cap on maximum hours spent in high-containment, supporting the development and release of the Occupational Safety and Health Administration (OSHA) infectious disease standards, creating a mechanism for registered entities to request SAR interpretations, ensuring that individuals with BSAT access can transfer access to another registered entity without the need to reapply, developing a process to relax FSAP requirements quickly in emergency situations, and developing a BSAT training program for customs inspectors.

Language requiring the documentation of BSAT drills and exercises was added to 42 CFR 73 sections 73.11(h), 73.12(e), and 73.14(f).^21^ Language requiring that BSAT trainees are instructed how to access the OIG hotline was added to 42 CFR 73 section 73.9(a)(7).^21^ Language requiring that responsible officials be included in a registered entity's biosafety and biosecurity committee was added to 42 CFR 73 section 73.11(f)(2).^21^ Language recommending that biosafety and biosecurity oversight committees involve all stakeholders in the development of relevant programs is included in the introduction of “Section II—Biological Risk” in the *BMBL*, but no language is currently included in the SAR.^24^

Language enhancing biosafety plan development guidance was added to 42 CFR 73 section 73.12(a).^21^ There is currently no language in the SAR suggesting a cap on the maximum hours an individual can spend in high-containment. However, there is Select Agent Gram from April 2016, which states that the FSAP will not issue new guidance on this topic but specifies that they support the recommendation.^28^ A maximum number of high-containment work hours may impede research efforts due to the unpredictable nature of biomedical research and laboratory staffing concerns, but this issue could be investigated under a new applied biosafety research program with sustained Federal funding.

The Biden Administration has announced plans to release a notice of proposed rulemaking for the OSHA infectious disease standards in October 2022 for public review and comment.^41,42^ A mechanism for individuals with BSAT access to transfer their access to another registered entity was implemented in July 2016.^40^ Language addressing expedited SRAs for approval to work with BSAT was present in 42 CFR 73 section 73.10(f) before the FTAC 5 recommendation, and the government health officials claim that current emergency waiver authorities are sufficient for anticipated needs.^21,40^ A BSAT training program for customs inspectors was developed and implemented in January 2016.^40^

### Necessary Number of High-Containment Laboratories in the United States

The necessary number of high-containment laboratories in the United States category contained a single recommendation from the FESAP that focused on identifying whether the United States had the number of high-containment laboratories necessary to protect against emerging infectious diseases while also minimizing the risk of disease due to accidental laboratory release. This recommendation reflects prior reports from the U.S. Government Accountability Office, which called for a review of the necessary number of high-containment laboratories needed nationally.^43,44^

The plan for this category included a Federal assessment of high-containment needs, an external assessment of high-containment needs, consideration of all assessments by the Federal government, and the development of a best practices checklist for use by registered entities when considering modifications to containment capacity. The Public Health Emergency's website claims that the Federal assessment of high-containment needs was performed, and that the assessment directly resulted in the creation of a best practices checklist for use by registered entities when considering modifications to containment capacity.^45^

However, it is unclear if the external assessment or consideration by the Federal government ever occurred. The original recommendation from the FESAP committee could be sufficiently satisfied if the results from the external assessment and Federal consideration were released. One of the concerns responsible for this recommendation is the presumption that more high-containment laboratory space could lead to more opportunities for a laboratory accident that results in a pandemic.^7^ Given the resources necessary to develop and maintain these facilities, planning is important to ensure adequate laboratory space for future needs.^7^

## Conclusion

The goal of this study was to analyze the progress in implementing the FESAP committee and FTAC recommendations through laws, guidance documents, and the development of trainings or programs. Of the 33 actions recommended by the 2 expert committees, 16 were addressed, 11 were insufficiently addressed, and 6 were not addressed. The results of this study show that more than half of the recommended actions were either not implemented or were insufficiently implemented.

The COVID-19 pandemic has provided a brief window of time where national attention is focused on issues surrounding biosafety and biosecurity.^8^ Steps should be taken now to update the SAR, secure funding for future research programs, educate the regulated community, and prepare for the next major pandemic before support for these issues fades. The 33 recommended actions were developed through a lengthy process that involved feedback from subject matter experts and comments from the public.^5–7^ All the recommendations should be fully implemented to enhance national biosafety and biosecurity while public officials are focused on the issue.^8^

The most important recommendations left unaddressed are to determine if the United States has sufficient high-containment laboratory space, develop and implement a sustained applied biosafety research program, develop an engaging bioethics training program, and create an ASRS-like BSAT incident reporting system for scenario-based training purposes.^7,23,35,36^ One major reason COVID-19 vaccines were able to be developed quickly was the years of prior research performed on SARS-1, MERS, and mRNA vaccine constructs.^46^ Similarly, it is crucial that the work of preparing our biosafety and biosecurity infrastructure for the next major pandemic begins today.
